# Key outcomes from stakeholder workshops at a symposium to inform the development of an Australian national plan for rare diseases

**DOI:** 10.1186/1750-1172-7-50

**Published:** 2012-08-10

**Authors:** Caron Molster, Leanne Youngs, Emma Hammond, Hugh Dawkins

**Affiliations:** 1 Office of Population Health Genomics, Department of Health, PO Box 8172, Stirling Street, Perth, 6849, Western Australia, Australia; 2 Centre for Population Health Research, Curtin Health Innovation Research Institute, Curtin University of Technology, Perth, Western Australia, Australia; 3 School of Pathology & Laboratory Medicine, University of Western Australia, Perth, Western Australia, Australia

**Keywords:** National plan, Rare diseases, Stakeholder consultation

## Abstract

**Background:**

Calls have been made for governments to adopt a cohesive approach to rare diseases through the development of national plans. At present, Australia does not have a national plan for rare diseases. To progress such a plan an inaugural *Australian Rare Diseases Symposium* was held in Western Australia in April 2011. This paper describes the key issues identified by symposium attendees for the development of a national plan, compares these to the content of EUROPLAN and national plans elsewhere and discusses how the outcomes might be integrated for national planning.

**Methods:**

The symposium was comprised of a series of plenary sessions followed by workshops. The topics covered were; 1) Development of national plans for rare diseases; 2) Patient empowerment; 3) Patient care, support and management; 4) Research and translation; 5) Networks, partnerships and collaboration. All stakeholders within the rare diseases community were invited to participate, including: people affected by rare diseases such as patients, carers, and families; clinicians and allied health practitioners; social and disability services; researchers; patient support groups; industry (e.g. pharmaceutical, biotechnology and medical device companies); regulators and policy-makers.

**Results:**

All of these stakeholder groups were represented at the symposium. Workshop participants indicated the need for a national plan, a national peak body, a standard definition of ‘rare diseases’, education campaigns, lobbying of government, research infrastructure, streamlined whole-of-lifetime service provision, case co-ordination, early diagnosis, support for health professionals and dedicated funding.

**Conclusions:**

These findings are consistent with frameworks and initiatives being undertaken internationally (such as EUROPLAN), and with national plans in other countries. This implies that the development of an Australian national plan could plausibly draw on frameworks for plan development that have been proposed for use in other jurisdictions. The translation of the symposium outcomes to government policy (i.e. a national plan) requires the consideration of several factors such as the under-representation of some stakeholder groups (e.g. clinicians) and the current lack of evidence required to translate some of the symposium outcomes to policy options. The acquisition of evidence provides a necessary first step in a comprehensive planning approach.

## Background

It has been argued that government responses to the needs of people affected by rare diseases have been sub-optimal [1]. This is despite the cumulative prevalence of rare diseases; the severity of impact on many people living with rare diseases, including carers and families and on publicly funded services. To address the argued lack of response, and since rare diseases share many common features [2], calls have been made for governments to adopt a more cohesive approach to rare diseases through development of National plans [2-8]; including calls in Australia [9-13].

National plans for rare diseases are official strategic documents comprised of specific priorities, actions, budgets and timetables that have been endorsed by governments [6]. National plans are seen as vehicles that provide frameworks incorporating a coordinated “whole-of-government” approach to rare diseases, as opposed to a “piece-meal” approach. These plans outline a cohesive clinical, public health and disability service approach to rare diseases that addresses prevention, timely diagnosis, early intervention, appropriate access to treatments and rehabilitation [3,5]. Such plans do exist internationally and provide guidance on how current services can be better integrated and how the translation of new knowledge relevant to the care and overall well-being of people living with a rare disease can be better informed by epidemiology, health economics, research to inform health and disability service planning, the provision of and access to services and information and industry policy [6].

The adoption of national plans, with dedicated funding, first occurred in France [14,15] with the second French National Plan released in 2011 and National plans have been adopted in other European countries including Belgium, Bulgaria, Czech Republic, Germany, Greece, Luxembourg, Portugal, Romania and Spain [16]. The European Union has recommended that all member states should have a national strategy for rare diseases by 2013. In 2008 the European Project for Rare Diseases National Plans Development (EUROPLAN) was funded by the European Commission. The aim of EUROPLAN is to provide an evidence-based framework for developing national plans. It identifies models and strategies that have been effective for addressing rare diseases, and outlines recommendations for actions to include in national plans. EUROPLAN also contains a list of indicators for implementing and monitoring national plans for rare diseases.

At present, Australia does not have a national plan for rare diseases. There are policies and plans that address the health and social needs of people living with chronic diseases, at both the state and federal government level. While these align with some of the unmet needs among the rare disease community, they do not necessarily cater for the range of needs specific to people living with rare diseases such as (lack of) access to timely diagnosis, disease management and access to treatment and rehabilitation programs. [11] Recognition of the need for an Australian national plan for rare diseases has gained momentum in recent years. The first published Australian call for better co-ordination in rare diseases came from the general practice sector [11] followed more recently by the Australian Paediatric Surveillance Unit (APSU http://www.apsu.org.au) [10], a research organisation that facilitates the study of rare childhood diseases. In 2009 APSU, with a small grant from the Australia Research Alliance for Children and Youth (ARACY), established a working group of 24 members to draft an outline for a national plan [17]. This plan was modeled on the French national plan [14] and called for actions related to awareness, education, improved access to health care, research, information collection and dissemination, a peak national body and advocacy.

To progress a national plan for Australia an inaugural Australian Rare Diseases Symposium was held in Western Australia in April 2011. The Symposium was structured around a series of plenary sessions. These were each followed by workshops where stakeholders discussed issues they deemed relevant to the development of a national plan. Stakeholders within the rare diseases community include, but are not limited to, the people affected by rare diseases such as patients, carers, and families, clinicians, allied health practitioners, social and disability services, researchers, universities, advocates, patient support groups, funders, industry (e.g. pharmaceutical, biotechnology and medical device companies), regulators and policy-makers. All of these groups were represented at the symposium.

Previously we have published a summary of the symposium and some preliminary information on the outcomes of the workshop discussions, such as the endorsement to develop a national plan for rare diseases in Australia. [9] This paper describes in more detail the key issues, goals and actions that were identified by stakeholders for the development of a national plan. The aim of the paper is to be transparent about the outcomes of the stakeholder workshops, how these compare to the content of EUROPLAN and national plans already developed in other countries and how the outcomes might be integrated in the ongoing process of developing a national plan for Australia.

## Methods

A workshop was held following each plenary session of the symposium. The overall objective of these workshops was to discuss the information presented in the plenary sessions and identify key issues, goals and actions for the further development of an Australian national plan for rare diseases. The topics for the plenary and workshop sessions were: 1) The development of national plans for rare diseases; 2) Patient empowerment; 3) Patient care, support and management; 4) Research and translation; 5) Networks, partnerships and collaboration; and 6) Moving forward post-forum. Keynote speakers for the plenary sessions included local and international experts in each of these domains. (For the symposium program see http://www.raredisease.com.au)

Three workshops (covering the topics of plan development, patient empowerment and moving forward post-forum) involved participants working within broad stakeholder groups, these being a) patients, carers, advocates, representatives of patient support organisations; b) scientists, researchers, industry representatives; c) medical, health, social services; and d) policy-makers, regulators, academics. The primary rationale behind this approach was to provide an opportunity for stakeholders to meet other’s in their group and provide an initial environment where all stakeholders felt comfortable contributing to discussions. This was particularly for patients and carers who we perceived might initially be reluctant to express their views in front of ‘experts’ such as clinicians. The remaining workshops involved participants working across stakeholder groups, where we perceived cross-stakeholder discussions might be particularly useful (e.g. on the topics of research, translation, networks, partnerships and collaboration).

Participants selected which table they wanted to join to partake in discussions. Each table was setup for 8 but could accommodate a maximum of 10 members. The process for each workshop involved participants discussing issues related to the preceding plenary session. A set of broad discussion guidelines, based on principles of deliberative public engagement [18] were provided to participants and these included the demonstration of respect for each other, everybody having a chance to talk, active listening, being willing to provide arguments for and against personal positions on issues, willing to change personal positions, willing to work towards consensus on issues and willing to accept and document any persistent disagreements.

One participant on each table self-selected to scribe and complete summary sheets of the table discussions. These sheets requested feedback on key issues for stakeholders, including those specifically for government and short-term and long-term goals/actions. All the sheets were collected by the workshop organizers and immediately following each workshop session, these were placed on a Feedback Board that was accessible to all symposium participants, enabling the broad sharing of discussion outcomes. The feedback sheets were used as data sources for the findings reported in this paper. One author (CM) conducted initial thematic analyses of the feedback, first within each workshop session and then across workshop sessions. The thematic outcomes of these analyses were then reviewed by all other authors and are presented in this paper.

## Results

Table 1 shows the geographical distribution of the participants; the majority of symposium participants were from Australia and the majority of those not from Australia were attending as keynote speakers. The stakeholder group with the greatest number of representatives at the symposium was people directly affected by rare diseases including patients, parents and carers, some of whom were formally representing patient support groups.

**Table 1:** Selected characteristics of symposium participants

**Characteristics**	**Participants (N)**
Type of stakeholder	
Patient, parent, carer, support group representative	66
Researcher (non-industry)	33
Clinician/other health professional	22
Industry	21
Government	16
Other	2
Country	
Australia	144
New Zealand	7
Europe	5
North America	2
Asia	2

Symposium participants identified a range of key issues for consideration in an Australian national plan for rare diseases, and each key issue was associated with a number of goals and actions. The key issues, goals and actions are summarized below under these six major headings: 1) National Plan; 2) Collaboration; 3) Information; 4) Services; 5) Funding.

### National plan

The key issue identified by stakeholders was an agreement that Australia must progress the development of a national plan for rare diseases. To this end symposium participants agreed that a set of guiding principles and recommendations, drawn from the outcomes of the symposium workshops, should be used to inform and frame the development of a national plan. Symposium attendees proposed that an Australian national plan for rare diseases should be founded on a human rights approach which promotes equitable access to health care for all. The plan should focus on the common issues that exist across the range of rare diseases and stakeholders. It should be sustainable, actionable, and aligned with other national strategies (e.g. disability, preventive health, chronic disease). The view of participants was that stakeholder input to the development of a national strategic plan for rare diseases should be guided and coordinated by a committee that is inclusive of all stakeholder groups. The strategic planning process should have Federal/State government agreement and commitment to action, be supported by national legislation^a^ and include processes for inclusive, continuing stakeholder consultation and engagement, which should inform and lead to the development of an implementation plan with timelines and processes for the monitoring, evaluation and review of the national plan’s goals and outcomes.

### Collaboration

The key issues identified in the area of collaboration were:

• A national, umbrella organisation should be established for rare diseases in Australia using best practice principles of governance. The organisation should be inclusive of all stakeholders in the rare diseases sector.

• Strong stakeholder networks are required national and internationally.

### Peak body

Symposium participants were universal in their support for the development of a national umbrella organisation for rare diseases. This was perceived to provide a national peak body and inclusive network that could undertake roles and functions such as the following; provide a single united voice for rare diseases; identify common issues and significant unmet needs of the rare diseases community; develop policy and position papers; raise public awareness; coordinate activities and events at a national level (e.g. Rare Disease Day); administer and coordinate a central “one-stop” website for information and educational materials; advocate, lobby and provide advice to government agencies, policy makers and politicians on the needs of the rare disease community; establish links with similar organisations internationally; set up and administer a national rare diseases registry; and provide a central hub to help co-ordinate and facilitate links amongst organisations at local, state, national and international levels.

A potential challenge identified by the workshop participants was how to establish the structure of a peak body. In particular, how to establish a core leadership group around which all stakeholders could unite as the ‘single voice’ for rare diseases and thereby promote a coordinated, national approach.

### Networks

The need for collaboration and networking was strongly expressed. In particular, symposium participants recommended that a formal, overarching Australian network for rare diseases should be established, similar to the European Organization for Rare Diseases (EURORDIS) and the U.S. National Organization for Rare Diseases (NORD). At the same time, existing networks should be recognized and valued (e.g. international, national and state based support groups and disease-specific coalitions such as TREAT-NMD). Greater collaborations and functional linkages were also seen to be required within and across Federal, state and local government departments and jurisdictions (e.g. health, finance, commercial services, transport, disability, employment). It was perceived that this might reduce “red tape” and enable more efficient and effective allocation of funding and utilization of existing resources.

According to symposium participants, collaborations and networks should be premised on open communication and the equal and inclusive engagement of all stakeholders, with the voices of patients, carers and families at the core of all decision-making processes. Strategies may be required to develop stakeholder solidarity, identify common ground, and reach consensus. Potential issues identified by participants included the presence of “territorialism” and “silos”, conflicting interests and expectations and the need to ensure that individual stakeholders (particularly disease-specific support organisations) are able to maintain their own identity.

### Information

The key issues identified in terms of information, data and evidence were:

• A standard definition of “rare diseases” is required for Australia. Preferably this would have international agreement.

• Publicly available information on rare diseases is needed for patients, families, health professionals, researchers, policy makers and other stakeholders.

• Awareness, education and engagement programs are required for the general public, health and other service professionals, government agencies, politicians and all other stakeholders.

• Politicians, governments and research bodies need to be lobbied in a coordinated way that is empowering for people affected by rare diseases.

• Australian evidence is needed to inform strategic planning, funding allocation decisions and research.

• Australian-based registries are required for rare disease groups and the rare disease community overall.

• Audits are required to identify “the current situation” in Australia with regards to rare diseases and “good practice models”.

### Public information

Symposium attendees identified the need for publicly available information on rare diseases to be easily accessible, centralized (e.g. a “one-stop shop” web portal/website or joining Orphanet and developing Australian and New Zealand specific content pages to link to this site), concise, reliable, accurate, up-to-date, easy to understand, and disability “friendly”. It was suggested that the types of information that should be easily accessible include: disease etiology; treatment and management options; a directory of specialists; local medical and social services; entitlements (e.g. financial); and support groups.

The need for increased public awareness, education and engagement programs was consistently suggested by symposium participants. These programs were perceived to offer outcomes that included; a raised profile of rare diseases as an aggregate category of diseases with a range of common issues; awareness of the rights and stereotypes of people affected by rare diseases; and recognition of the impact of rare diseases as a significant public health issue and priority area for State and Federal government support and funding.

### Lobbying

Lobbying and the dissemination of information to politicians and the public service was seen to be crucial to achieving several outcomes, including; bi-partisan political support; government recognition of the personal, social and financial impact of rare diseases on families; recognition of economic costs and need for planning; and recognition of rare diseases as a national health and research priority area. Engagement with professional lobbyists and high profile sponsors, opinion leaders and “champions” in business and government was identified as important for effective lobbying. At the same time patient and carer champions and ambassadors should be supported and enabled to advocate for themselves.

### Australian research and evidence

Symposium participants indicated Australia needs a national framework for research on rare diseases. They also suggested that Australian-based researchers and research institutions across academia, clinical practice, government and industry must collaborate and work together to be most effective in conducting multi-disciplinary research on rare diseases (e.g. identifying sufficient cases for clinical trials). Patients, carers and families should be directly involved in decisions about research on rare diseases. Suggested priority areas for research included: quality of life and social impact studies (e.g. needs of patients/carers); epidemiology and health economics (e.g. prevalence of rare diseases, burden of disease, cost-effectiveness of treatments and health services); and clinical trials (e.g. drug trials, novel therapies). Translating research into better prevention, early intervention, diagnosis and treatment to improve health and quality of life outcomes was also identified as a priority. It was suggested that this requires the integration of research activity within the clinical context.

In Australia the National Health and Medical Research Council (NHMRC) is the peak agency for funding health and medical research. Symposium participants suggested that the NHMRC must recognise the value of research on rare diseases and allocate funding accordingly. Public/private collaborations and funding mechanisms should also be explored. Stakeholders also suggested that data collection by governments (State and Federal) be standardized so that information can be exchanged and aggregated across jurisdictions. Participants identified a range of potential barriers in relation to data sharing, including ethical issues and policy requirements for health systems in different jurisdictions, privacy and confidentiality issues inherent in data linkage and ownership of the aggregated data. Stakeholders expressed that, in areas such as clinical trials, bureaucracy needs to be minimized and the co-ordination of multi-institutional and/or multi-State research needs to be improved, including uniform ethics approval processes. Similarly, novel approaches to research may be required, and should be supported by funding bodies. For example, the low prevalence of most rare diseases means that the placebo/double blind trial design required by regulatory bodies is usually not possible and innovative trial designs should be recognized for rare diseases. Finally, stronger research training, career pathways and the attraction and retention of research expertise are required.

### Audits

A range of “audits” were recommended as a means of collating information on the current situation in Australia to identify: common issues and unmet needs across rare diseases; the full range of stakeholders; key individuals and organisations; existing expertise and perceived gaps in expertise; existing networks, partnerships, clinical and other services, programs and systems; registries (disease groups with and without them); research and research facilities (including clinical trials); funding mechanisms; information, education and training resources; and challenges for people affected by rare diseases, in particular, those who live in rural and remote areas of Australia. It was perceived that strategic plans should be developed and implemented to address any gaps and inefficiencies as well as identifying opportunities to link existing agencies, organisations, groups and programs to achieve synergies and capture economies of scale.

### Services

The key issues identified in the areas of services and support for people affected by rare diseases, were:

• All patients, carers and families should have equal access to programs and services.

• A generic (overarching) model of care (or clinical pathway) for rare disease is required. ^b^

• Streamlined, coordinated whole-of-lifetime service provision is required.

• A case co-ordination approach is required that encompasses all services relevant to patients, carers and families affected by rare diseases. This includes health (e.g.allied health, clinicians, medical specialists, GPs etc.), education, disabilities, employment, local community services and social support services.

• Diagnosis needs to be early, accurate and accessible for all Australians.

• Effective, direct communication and information sharing across health services and health professionals (e.g. GPs, specialists); and between health professionals and patients, carers and families is critical to quality of life and clinical care outcomes.

• Health professionals need more awareness and support in relation to rare diseases. This is particularly for GPs but also for medical students, specialist clinicians and allied health professionals

• Patients, carers and families affected by rare diseases need support.

• Existing support groups should be strengthened and new groups established for diseases that currently do not have a support group.

### Coordinated whole-of-lifetime care

Symposium participants expressed the view that there should be no gaps in service delivery across the lifespan of an individual; programs and services should seamlessly span transitions from pediatrics to adulthood to aged care. To facilitate this, it was suggested that case coordinators are needed who will be the first point of contact for patients, carers and families. Case coordination should: have an individualized needs-driven focus (not a disease focus); emphasize flexibility, integration, cost effectiveness and cost efficiencies for the health system; and be founded on national best practice standards and/or model of care for rare diseases. It was perceived that a generic model(s) of care could focus on problems common to many types of rare diseases; and address all aspects of care including diagnostic services, treatment options, multi-disciplinary communication and service integration. It was also perceived that multi-disciplinary clinic(s) could facilitate case coordination. Such centers could undertake various roles including; establishing and managing clinical networks; acting as reference centers and centers of excellence that provide diagnosis, treatment, advice, follow-up care, information and entitlements in a coordinated cross-jurisdictional (e.g. state-state, state-national) manner.

All workshop groups expressed the view that patients must have fair and equitable access to services, programs and treatments (including novel, innovative and/or trial treatments), and that the availability of therapies and genetic testing must be improved. Specifically, gaps in service delivery were perceived to exist across States and for those in rural and remote areas. It was suggested that genetic testing for rare diseases should be covered by Medicare (a national government initiative that delivers programs such as the Australian Pharmaceutical Benefits Scheme) and support should be provided for “not-yet-diagnosed” patients and families. The symposium participants also suggested the system for determining the cost of and reimbursement for orphan drug treatments needs to be changed. Some suggested options include: using a different approach, or alternative pathway, to determine access to rare disease drugs, rather than the existing Life Saving Drugs Program or Pharmaceutical Benefits Scheme; introduce a Rare Diseases Act; and/or identifying the Australian community’s values regarding access to orphan drugs and how much, as taxpayers, they are willing to pay for medicines to treat those living with a rare disease.

### Information sharing

Within the context of service delivery, symposium participants held the view that patients, carers and families need access to information from health professionals that is up-to-date, accurate, reliable and easy to understand. At the same time, health professionals should keep an open mind about therapies and treatments which may impact on rare diseases and listen to patients’ stories about what works. The knowledge and expertise of patients, carers and families should be acknowledged and respected. System(s) are also required that will enable the sharing of clinical and other health information within and across health services, health professions and other relevant service providers (e.g. disability services). Examples of suggested systems include e-health records, a privacy-protected website that records patient details and recommended/adopted clinical pathway approaches and a central repository of patient action plans. Greater sharing of service-user information would further enable more effective case coordination and whole-of-lifetime service delivery.

### Support for health professionals

Health professionals are perceived to need greater support to provide effective services for those affected by rare diseases. Examples of suggested approaches to support include: the provision of resources and tools (e.g. referral/diagnostic tools for GPs, education programs); primary health care partnerships; national training packages; rare diseases being included in medical training curriculum; standardized protocols for the delivery of clinical information and better awareness of and access to information (e.g. Orphanet).

### Support for people affected by rare diseases

Patients, carers and family members are perceived to need improved support services. It was suggested that the types of support may include: support groups and local contacts; medical aids; education/information; employment and alternatives to employment; supported accommodation; respite services; mental health and psycho-social support; and changes to local government policies (e.g. car parking, physical access to services).

Participants suggested that support groups should be valued and assisted, either financially or in-kind, by other stakeholders. This was in a range of areas such as funding, best-practice governance, business management, strategic planning, marketing and fundraising. It was also indicated that support groups should be accredited. This would require support groups to meet best practice standards for governance and business management.

### Funding

Symposium participants proposed the need for funding to be sustainable and patient-focused, that is, funding should address the needs of patients, carers and families affected by a rare disease. A range of sources of funding and infrastructure should be considered (e.g. government, industry, community groups, not-for profit groups, philanthropists, and public-private partnerships between academia, industry, government and community groups).

Improved funding was perceived to be critical to the development and implementation of a national plan on rare diseases. Symposium participants suggested funding is required in a range of areas such as:

• Research, for example: to provide incentives for national and international collaboration; incentives to encourage industry (e.g. marketing exclusivity; research scholarships; seed funding to start research projects; translational research; health economic studies; quality of life studies and reimbursement of patients’, carers’ and family research costs).

• Infrastructure, for example: for research; enabling grants; to build critical mass and to establish registries

• GP services, for example: to provide GP incentives to support patients/families with complex needs.

• Clinical and other services, for example: genetic testing and diagnostic services; care and treatment planning; incentives to take on patients/families with complex needs; training positions and specialists in public hospitals.

• Support groups and organizations, for example: seed capital to fund project officers; ongoing funding for an umbrella organisation; administration support; conference attendance and website development.

## Discussion

The views expressed by participants at the inaugural Australian Rare Diseases Symposium indicate widespread support for a national plan for rare diseases in Australia, among a range of stakeholder groups in the rare diseases community, including patients, carers, clinical and disability services, academia and the biotechnology/pharmaceutical industry. There also appears to be general agreement on a range of key issues, goals and actions for inclusion in a national plan, including the need for a peak body for rare diseases, networks of stakeholders, Australian research and evidence on rare diseases, public information and education and accessible, integrated services.

The outcomes of the symposium workshops (i.e., key issues, goals, actions for a national plan) are consistent with the findings from an earlier stakeholder survey conducted by the National Rare Diseases Working Group (NRDWG) convened by the Australian Pediatric Surveillance Unit (APSU) [10,13]. They are also consistent with findings, frameworks and initiatives being undertaken internationally (such as EUROPLAN), and with the aims and priorities of national plans in other countries including Belgium, Bulgaria, Czech Republic, Germany, Greece, Luxembourg, Portugal, Romania and Spain^c^[6]. This is in areas including mechanisms for facilitating patient empowerment, such as a peak body [19,20] and web-based information and networking resources [19,21-23]; the need for reliable epidemiological data; the need for registries to facilitate clinical research, public health surveillance, and service/program evaluation [24]; and the need for research infrastructure, including partnerships and collaborations, research sites with common protocols and multi-disciplinary research teams [3,5,7,25-31]. Similar to stakeholders in other countries, symposium participants acknowledged the complexities of implementing some of these actions. This is particularly in the area of research, with issues such as small patient populations meaning constraints in epidemiologic research and low -powered clinical trials; limited clinical data and biological specimens, which limits studies of disease causes and mechanisms; a low interest from the pharmaceutical industry due to a limited market incentives (low return on investment); and a limited number of experts and clinicians committed to the study of rare diseases [32].

There also appear to be similarities with other countries with respect to accessing timely, correct diagnosis; integrated diagnostic pathways, case-coordination of multi-disciplinary care; national centers of excellence; clearly defined diagnostic guidelines; quality information; awareness among health professionals of rare diseases; support for carers; and treatments including orphan drugs [1,19,26,33-38]. A key implication, of the workshop outcomes being consistent with international approaches to rare diseases, is that in developing an Australian national plan it seems plausible to draw on frameworks for plan development that have been proposed for use in other jurisdictions, such as EUROPLAN. This might enable planners in Australia to benefit from the experience of those who have already developed national plans and minimise any unnecessary re-inventing of the wheel (a preferred outcome expressed by stakeholders at the symposium).

The translation of the symposium outcomes to government policy (i.e., a national plan) requires the consideration of several factors. Firstly, while a range of rare disease stakeholders were represented at the symposium, it should nonetheless be acknowledged that some stakeholder groups (e.g. clinicians) appeared less well represented than other groups (e.g. support group members, researchers). The symposium findings are thus limited, to the extent that those not present at the symposium may hold different views from those views expressed by the symposium participants. Hence, in the assessment of the unmet needs of the rare diseases community and as jurisdictions consider the development of a national plan, it will be important to monitor which stakeholder groups engage most strongly and develop strategies and a range of mechanisms for encouraging participation by all stakeholders. These might include, for example, online discussions, written submissions, in-depth interviews, surveys and forums. Ongoing, systemic stakeholder engagement in the planning process, as identified by the participants in the symposium engagement, will be critical to the successful development and implementation of an Australian national plan for rare diseases.

Secondly, some issues and goals identified by symposium participants could be viewed as “motherhood” notions, in that little detail was provided as to how the issue or goal might be implemented, or what barriers or limitations to implementation might exist. For example the implementation of integrated, coordinated care and multi-disciplinary teams would likely require the consideration of a range of challenges, which in our view need to be explored before such systemic changes could be put forward as part of a national plan on rare diseases. As noted by several authors [39,40] evidence-based policy options are required, along with information on the scope of challenges, barriers to and potential positive outcomes of integrated, coordinated care and what methods would be most effective for measuring the outcomes of such healthcare delivery. Further the concept of integration is contested, so clarification is needed of the terminology and the meaning attributed by stakeholders to the concept. Specifically, further investigation is required into what integration would look like from each stakeholder’s point of view, including the patient, clinical practice, public health and disability service perspectives.

The literature notes that other issues also require greater clarity of terminology including the concept of ‘rare diseases’ itself. A European study indicates the term does not have a common meaning across all stakeholders and is meaningless for clinicians [41]. Making assumptions that all stakeholders have similar understandings of the symposium outcomes, simply because they used the same terminology to arrive at those outcomes, might be problematic for the development of a national plan. One starting point for the standardization of terminology is the adoption of an internationally consistent definition of rare diseases. To this end the following definition adapted from the European Commission Directorate General for Health and Consumers Public Health policy, ^d^ and more recently used by European countries developing their national rare disease plans, [42] has been proposed as the definition in Australia:

A rare disease is any disorder or condition that is a life-threatening or chronically debilitating disease which is statistically rare, with an estimated prevalence of less than 5 in 10,000 or of similarly low prevalence and high level of complexity that special combined efforts are needed to address the disorder or condition.

## Conclusions

In Australia several of the components relevant to a national plan for rare diseases are already in existence or being established. For example policy and planning is in place for chronic diseases and disability services, people with rare diseases utilize genetic and other clinical services and specializations, and a number of patient registries, biobanks, bioinformatics and genetic technologies are operational. Notwithstanding this there are a number of areas which if better understood and coordinated into a specific national plan might bring great benefits to the rare disease community. Planning in the rare disease setting is also likely to translate to benefits for tackling common multi-genic disorders among the broader chronic disease community including advances in pharmacogenomics (personalised medicine) [43-45]. To progress national planning we believe some form of prioritization, of the key issues, goals and actions identified by symposium participants, is required. In our view, conducting evidence-gathering activities, such as audits of the current situation regarding rare diseases in Australia, is a good starting point for filling gaps in knowledge and achieving other goals and actions identified by symposium participants. In line with the views of other authors [3-5,29-31,46,47] we contend that the acquisition of evidence provides a necessary step in a comprehensive planning approach.

## Endnotes

^a^The use of the terms generic and model of care and clinical pathways require definitions, but was meant to indicate the need to have a map or plan for the process of diagnosis, follow-up counselling, treatment etc.

^b^The use of the terms generic and model of care and clinical pathways require definitions, but was meant to indicate the need to have a map or plan for the process of diagnosis, follow-up counselling treatment etc.

^c^http://ec.europa.eu/health/rare_diseases/national_plans /detailed/index_en.htm.

^d^http://ec.europa.eu/health/rare_diseases/policy/index_ en.htm.

## Competing interests

The authors declare that they have no competing interests.

## Authors’ contributions

All authors made substantial contributions to the conception and design of the workshop methodology, acquisition of data, interpretation of data, drafting of the manuscript or critical revision and have given final approval of the version to be published.

## Authors’ information

National Rare Diseases Coordinating Committee Members: H Dawkins (Chair), E Elliott (Deputy Chair), M Fookes, J Forman, J Goldblatt, J McGaughran, C Molster, K North, S Peden, H Stevens and Y Zurynski.

## 

National Rare Diseases Working Group Members: N Barker, G Baynam, S Broley, S Chilvers, M Crosthwaite, H Dawkins (Chair), M Evans, C Franklin, C Molster, L Murphy, S O'Sullivan, S Shapland and M Stafford.
